# Diagnostic Performance of Radiomics Modeling in Predicting the Human Papillomavirus Status of Oropharyngeal Cancer: A Systematic Review and Meta-Analysis

**DOI:** 10.7759/cureus.82085

**Published:** 2025-04-11

**Authors:** Derek Vos, Noah Yaffe, Claudia I Cabrera, Nicole M Fowler, Brian D D'Anza

**Affiliations:** 1 Otolaryngology, Case Western Reserve University School of Medicine, Cleveland, USA; 2 Otolaryngology - Head and Neck Surgery, University Hospitals Cleveland Medical Center, Cleveland, USA

**Keywords:** artificial intelligence, hpv-related oropharyngeal cancer, human papillomavirus, machine learning, radiomics

## Abstract

In this review, we sought to assess the diagnostic performance and methodological quality of studies utilizing radiomics for the prediction of human papillomavirus (HPV) status in patients with oropharyngeal squamous cell carcinoma. A comprehensive literature search of PubMed, Ovid, Cochrane, Web of Science, and Scopus from inception until June 7, 2022, was performed to identify eligible studies. Strict inclusion and exclusion criteria were applied to the identified studies. Data collection was performed by two independent reviewers with disagreements resolved by consensus review with a third reviewer. In total, 14 articles were chosen, with a total of 15 radiomics models. Of the included studies, 12 models reported sensitivity, with a mean of 0.778 (standard deviation (SD) = 0.073). Similarly, 12 models reported specificity, with a mean of 0.751 (SD = 0.111). The area under the curve (AUC) was reported by all 15 models, with a mean of 0.814 (SD = 0.081). Finally, accuracy was reported by eight models, with a mean of 0.768 (SD = 0.044). A meta-analysis was performed on eight studies that reported AUCs with confidence intervals (CIs), returning a pooled AUC of 0.764 (95% CI = 0.758 to 0.770). The Radiomics Quality Score (RQS) was applied to each included study as a measure of quality. RQS ranged from -1 to 22, with a mean of 13.4 and an intraclass coefficient of 0.874. Radiomics modeling has shown promise in serving as a diagnostic indicator for HPV status in patients with oropharyngeal cancer. Nevertheless, the quality of research methodologies in this area is a limiting factor for its broader clinical application and highlights the need for enhanced funding to support further research efforts.

## Introduction and background

Oropharyngeal carcinoma has been on the rise despite overall decreases in tobacco use. This rise is due, in large part, to the increase in human papillomavirus (HPV)-positive oropharyngeal carcinoma. In fact, HPV-positive oropharyngeal cancer with non-keratinizing squamous cell carcinoma histology is estimated to account for 30-60% of all oropharyngeal cancers [1-3]. Furthermore, HPV-positive oropharyngeal carcinoma is classified by the National Comprehensive Cancer Network as a separate entity from HPV-negative carcinoma, with its own distinct staging and treatment guidelines [4,5]. Therefore, determining the HPV status of oropharyngeal carcinoma is a key element in the management and prognostication of such patients [6,7].

Currently, testing for HPV status is via p16 immunohistochemistry, which requires an invasive procedure to obtain a tissue sample. This adds both cost and risk of complications [8,9]. Despite tissue sampling and p16 immunohistochemistry representing one of the most common methods used to determine HPV status, there is benefit in considering non-invasive options. As such, new methods of distinguishing HPV status in this patient population are an area of increased research interest.

**Figure 1:**
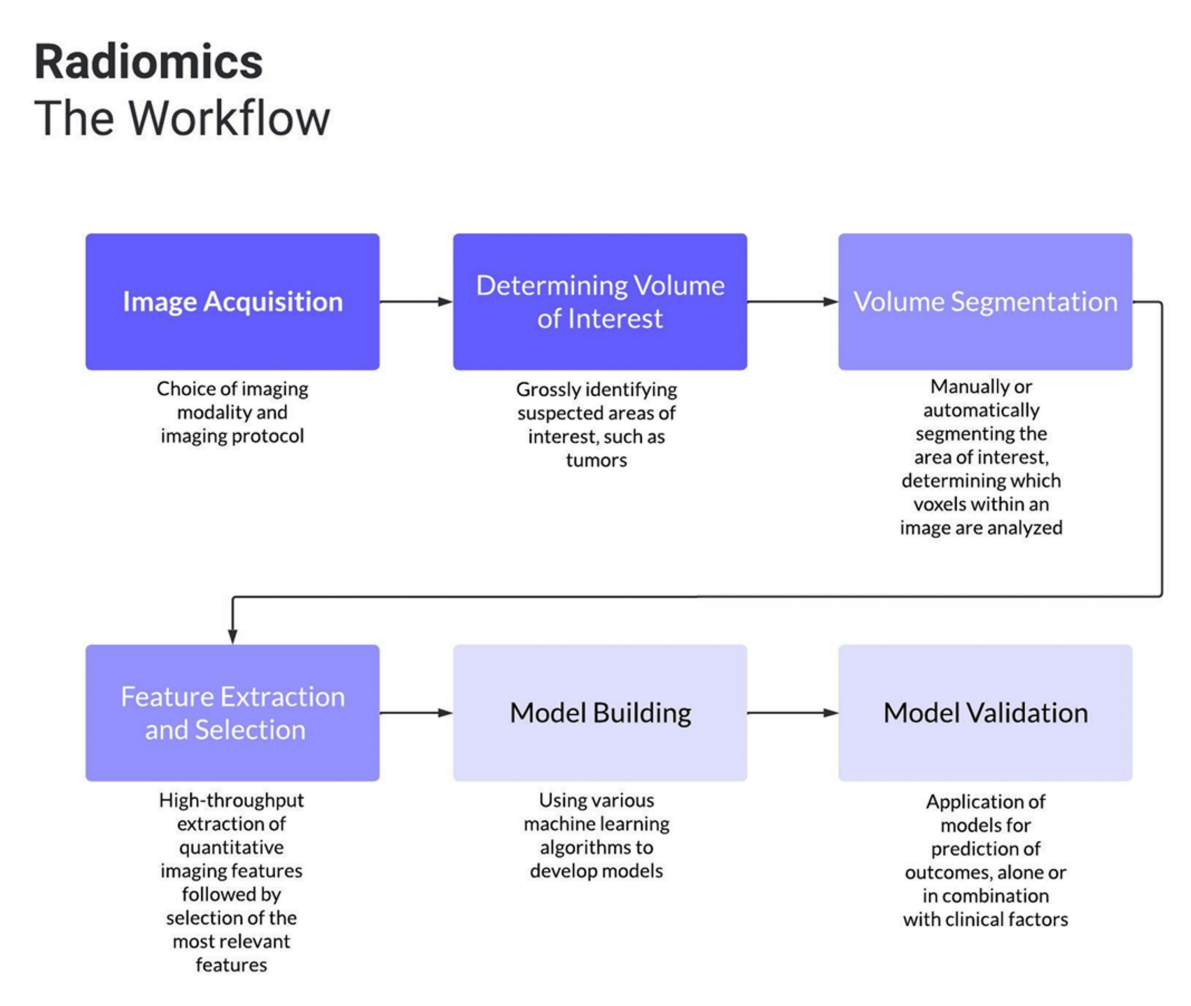
Radiomics workflow. Flowchart demonstrating the basic radiomics workflow. Authors’ own image.

One such possible method in determining HPV status is radiomics, a branch of artificial intelligence that uses high-throughput (ability to efficiently process a large volume of information) extraction of quantitative imaging features to develop predictive models. For example, radiomics models may be able to identify CT textures or other features that are correlated with HPV status that the radiologist is unable to. The primary advantage of using a radiomics framework over other more traditional methods of determining HPV status is that it offers a non-invasive approach, whereby baseline medical imaging used in the initial diagnosis of oropharyngeal cancer can be leveraged to predict HPV status. The basic radiomic workflow can be broken down into the following five distinct steps and is shown in Figure 1: (1) image acquisition, (2) determining volumes of interest, (3) segmenting these volumes, (4) extracting and qualifying descriptive features from segmented regions, and (5) developing models for predicting outcomes [10-13].

The potential benefits of the radiomics framework have been illustrated in several other cancers, such as hepatocellular carcinoma, breast cancer, and glioma, demonstrating effectiveness in predicting molecular subtype, prognosis, or therapy response [14-17]. Currently, several studies have been published that examine the radiomic features of oropharyngeal cancer patients that are associated with HPV status and the ability to predict HPV status based on these features [18]. However, model performance differs based on the originating institution and the sample used in the study. Given these potential challenges with using radiomics models to predict HPV status in oropharyngeal cancer patients, a review of the current state of the literature is needed to assess the clinical utility of such a technique. Therefore, we conducted a systematic review and meta-analysis with the primary aim of assessing the diagnostic accuracy and predictive value of radiomics in predicting HPV status of patients with oropharyngeal carcinoma. We further intended to assess the methodological quality of radiomics studies used in the prediction of HPV status in these patients.

## Review

Methodology

This meta-analysis was conducted following the Preferred Reporting Items for Systematic Reviews and Meta-Analyses (PRISMA) guidelines. The study was prospectively registered at the PROSPERO website with the registration number CRD42022337055 on June 22, 2022.

Search Strategy

A search of the databases PubMed, Ovid, Cochrane, Web of Science, and Scopus from inception to June 8, 2022, restricted to English, was conducted to identify related eligible studies. In brief, this search consisted of terms related to machine learning algorithms and radiomics, as well as HPV status and oropharyngeal squamous cell carcinoma. The full search details may be found in the Appendices.

Eligibility Criteria

Strict inclusion and exclusion criteria were applied to the studies identified using the previously described search strategy. The inclusion criteria consisted of the following: (1) population: patients with oropharyngeal squamous cell carcinoma and confirmed HPV status; (2) intervention: machine learning algorithms applied to pre-treatment imaging data for the prediction of HPV status; (3) outcome: diagnostic performance of the radiomics model, assessed using sensitivity, specificity, and the receiver operating characteristic (ROC) area under the curve (AUC) with their respective confidence intervals (CIs); and (4) design: retrospective or prospective original research studies in which HPV status prediction was included as an outcome measure. Importantly, to be included in the meta-analysis, identified studies were required to have reported AUC with CIs. Studies were excluded from our analysis based on the following exclusion criteria: (1) case reports, review articles, letters, conference abstracts, and editorials; (2) studies in which ultrasound was the imaging modality utilized to create and assess radiomics models; (3) studies with duplicate patients and data; and (4) studies in which the clinical utility of radiomics models in predicting HPV status was not a primary goal. The identified articles were then screened by title and abstract, followed by full-text review of those deemed potentially eligible. This review was performed independently by two reviewers (DV, NY), with differences resolved by consensus review with a third reviewer (CC).

Data Collection

Before data collection, a comprehensive data sheet was prepared to be used in extraction. The variables identified included (1) study characteristics, including author, publication year, study type, study design, and single- vs. multi-center; (2) patient characteristics, including sample size, tumor site, tumor stage, mean age, smoking status, and HPV status; (3) radiomics model characteristics, including image modality, use of contrast, pre-processing status, segmentation method and software, type and method of validation, method of feature selection, number of features included in the final model, and modeling method; and (4) radiomics model performance, including sensitivity, specificity, and AUC. This data was collected independently by two reviewers (DV, NY), with consensus review performed by a third reviewer with expertise in radiomics and machine learning (CC). Data is available on reasonable request to the authors.

Assessment of Study Quality

The methodologic quality of the radiomics studies was rated independently by two reviewers using the Radiomics Quality Score (RQS), a commonly utilized tool within the radiomics literature to assess quality [19]. Inter-rater reliability was determined using the intraclass coefficient (ICC).

Statistical Analysis

Following full-text review and data collection, a meta-analysis was performed on studies with sufficient data regarding model performance. Due to the inconsistency of effect measures and to improve the generalizability of the results, we used a random effects model due to moderate-to-high heterogeneity in the articles. Data was analyzed for AUC for studies that reported 95% CI. AUCs were pooled in random effects and heterogeneity of studies was assessed using Cochran’s Q test, as well as Higgins’ inconsistency index (I^2^). Publication bias was assessed using Egger’s test for a regression intercept and Begg’s test for rank correlation, as well as Deeks’ funnel plot. All analyses were performed in MedCalc® Statistical Software version 22.001. All results were presented as effect estimates with 95% CI.

Results

Search Results

**Figure 2:**
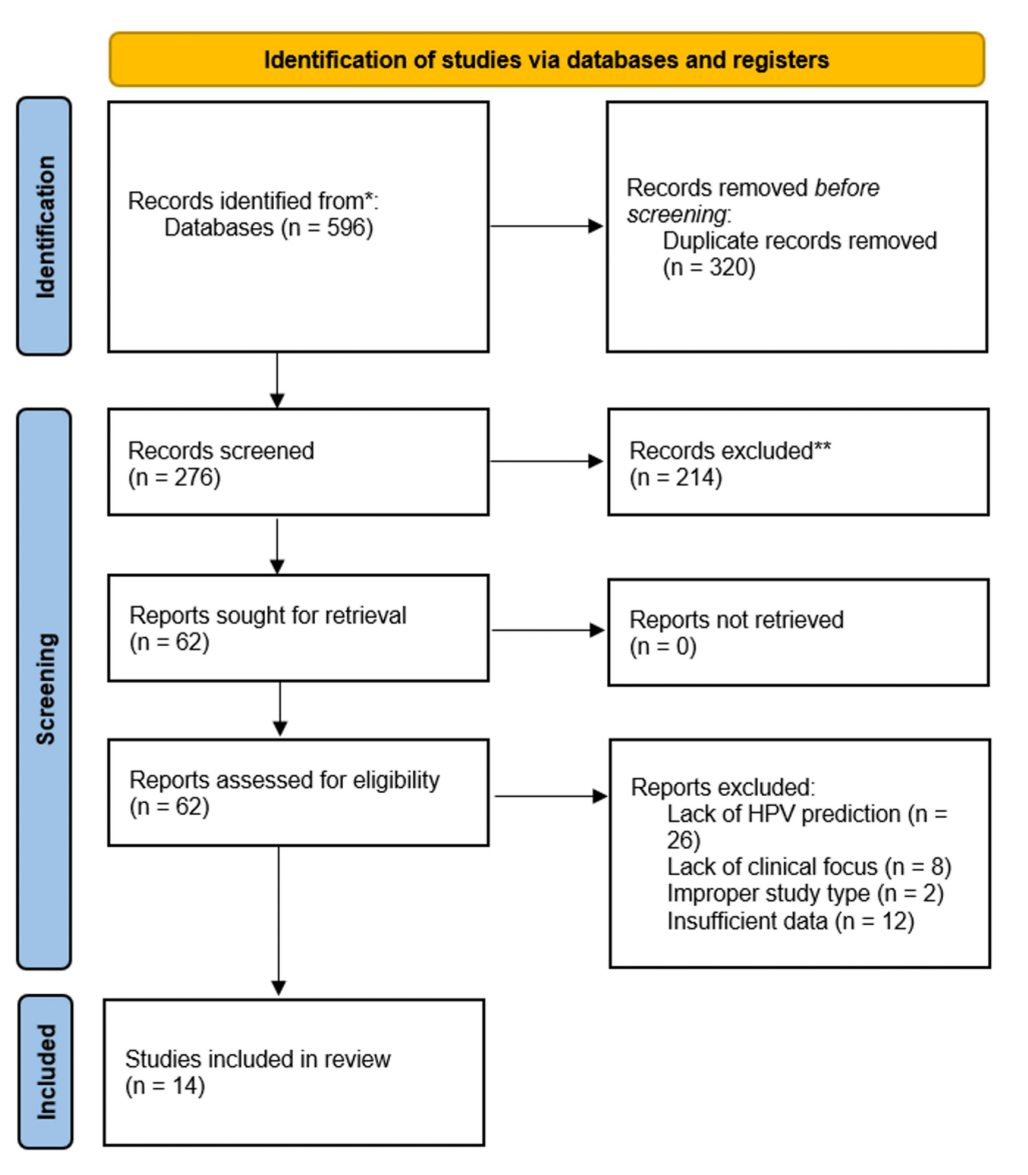
Article screening and inclusion per the Preferred Reporting Items for Systematic Reviews and Meta-Analyses (PRISMA) guidelines. *: Databases searched included PubMed, Ovid, Cochrane, Web of Science, and Scopus. **: Records excluded after abstract review.

A total of 276 studies were identified using the previously described search strategy after removal of duplicates. Following title and abstract screening, 62 articles were identified for full-text review and comparison against inclusion/exclusion criteria. Ultimately, the final selection consisted of 14 articles, with one article including both a two-dimensional (2D) and three-dimensional (3D) radiomics model which were separately evaluated [20-33]. The details of the search process can be found in the PRISMA flow diagram shown in Figure 2.

Review of Included Studies

Characteristics of the 14 studies that were included in this study can be found in Table 1 and Table 2. All but one of the included studies were retrospective studies, with each study being published between 2017 and 2021. The majority of studies were single-center studies (n = 10, 71.4%). When reported, the most commonly identified tumor site was the oropharynx, and all studies included a segment of the oropharynx, with one identifying specific subsites [24]. A plurality of studies (n = 11, 78.6%) included patients with tumor stages T1-T4. Reporting of smoking status within the included studies was inconsistent, with only six (42.9%) studies including this information as part of their analysis.

**Table 1:** Study and patient characteristics. HPV: human papillomavirus; HNSCC: head and neck squamous cell carcinoma

Author	Publication year	Study type	Study design	Single/Multi-center	Sample size	Tumor site	Tumor stage	T1–T2	T3–T4	Mean age	Smoking	Non-smoking	HPV +	HPV-
Bagher-Ebadian et al. [31]	2021	Retrospective	Cohort	Single	128	Oropharynx	N/A	N/A	N/A	N/A	N/A	N/A	60	68
Bogowicz et al. [20]	2017	Retrospective	Cohort	Single	149	Oropharynx, hypopharynx, larynx, oral cavity	T1–T4	33	60	61.9	N/A	N/A	36	57
Bos et al. [21]	2021	Retrospective	Cohort	Single	153	Oropharynx	T1–T4	78	75	61	114	39	76	77
Choi et al. [22]	2020	Retrospective	Cohort	Single	86	Oropharynx	T1–T4	60	26	60.9	50	36	53	33
Haider et al. [29]	2020	Retrospective	Cohort	Multi	435	Oropharynx	T1–T4	166	160	60.59	N/A	N/A	244	82
Huang et al. [23]	2019	Retrospective	Cohort	Multi	166	Larynx, oral cavity, pharynx	T1–T4	30	83	60.1	41	73	21	92
Mukherjee et al. [24]	2020	Retrospective	Cohort	Multi	184	Non-cutaneous HNSCC	T1–T4	29	84	59.8	91	22	25	57
Ranjbar et al. [30]	2018	Retrospective	Cohort	Single	107	Oropharynx	T1–T4	54	53	60.15	N/A	N/A	92	15
Ravanelli et al. [25]	2018	Retrospective	Cohort	Single	59	Oropharynx	T2–T4	25	34	64.9	34	25	28	31
Ren et al. [26]	2020	Retrospective	Cohort	Single	47	Oropharynx	T1–T4	15	32	58.3	N/A	N/A	15	32
Sohn et al. [32]	2021	Retrospective	Cohort	Single	62	Oropharynx	N/A	N/A	N/A	58.7	N/A	N/A	39	4
Song et al. [27]	2021	Retrospective	Cohort	Multi	582	Oropharynx	T1–T4	89	91	62.7	65	115	100	80
Suh et al. [33]	2020	Retrospective	Cohort	Single	60	Oropharynx	T1–T4	31	26	59	N/A	N/A	48	12
Vidiri et al. [28]	2019	Prospective	Cohort	Single	73	Oropharynx	T1–T4	29	44	63.55	44	29	54	19

**Table 2:** Radiomics model characteristics. FDG PET/CT: 18F-fluorodeoxyglucose positron emission tomography/computed tomograpy; LASSO: least absolute shrinkage and selection operator

	Image modality	Contrast	Image pre-processed (yes/no)	2D/3D segmentation?	Manual or automatic segmentation?	Segmentation software	Internal or external validation	Internal validation method	Cross-validation	Feature selection method	Total Features	Modeling method
Bagher-Ebadian et al. [31]	CT	Yes	Yes	3D	Manual	Eclipse v11.5 TPS	Internal	Random	Yes	Point biserial correlation coefficient (rpb)	15	LASSO logistic regression
Bogowicz et al. [20]	CT	Yes	Yes	3D	Automatic, confirmed with manual	In-house developed radiomics software	Internal	Validation cohort was from a separate institutional study	No	Principal component analysis	4	Multivariable logistic regression
Bos et al. [21]	MRI	Yes	Yes	3D	Manual	N/A	Internal	Random	Yes	Interclass correlation coefficient + Pearson correlation	3	Multivariable logistic regression
Choi et al. [22]	CT	Yes	Yes	3D	Semi-automatic	syngo.via	External	N/A	No	Random forest	9	GLM
Haider et al. [29]	FDG PET/CT	Yes	Yes	3D	Semi-automatic	3D-Slicer	Internal and External	Random	Yes (20x five-fold cross-validation)	RIDGE	21	NBayes
Huang et al. [23]	CT	Yes	No	3D	Manual	Horos	External	N/A	Yes (nested stratified 10x10 CV)	Interclass cofficient + mRMR	5	Multivariable logistic regression
Mukherjee et al. [24]	CT	Yes	Unclear	2D	Manual	Horos (pencil tool of DICOM viewer)	External	N/A	Yes (10-fold cross validation repeated 10 times)	Principal component analysis (PCA)	29	Sparse logistic regression
Ranjbar et al. [30]	CT	Yes	Yes	2D	Manual	OsiriX	None	N/A	Yes (leave one out cross-validation)	Principal component analysis + Forward selection algorithm	N/A	Diagonal Quadratic Discriminant Analysis (DQDA)
Ravanelli et al. [25]	MRI	Yes	No	2D	Manual	TexRAD	None	N/A	No	No feature selection method - the model itself is the selection method	2	Multivariable logistic regression
Ren et al. [26]	CT	Yes	Unclear	2D	Manual	3D Slicer software	None	N/A	Yes (nested cross-validation with 10-fold inner and 10-fold outer loops)	Pearson correlation + Random Forest (RF) [this is what was used for the final model]	6	RF with SMOTE
Ren et al. [26]	CT	Yes	Unclear	3D	Manual	3D Slicer software	None	N/A	Yes (nested cross-validation with 10-fold inner and 10-fold outer loops)	Pearson correlation + random forest (RF) (this is what was used for the final model)	7	RF with SMOTE
Sohn et al. [32]	MRI	Yes	Yes	3D	Semi-automatic	Open-source software	Internal	Temporally	Yes (10-fold cross-validation with bootstrapping)	LASSO + logistic regression	6	LASSO logistic regression
Song et al. [27]	CT	Yes	Unclear	2D	Manual	Hand-annotation tool	Internal and External	Random	Yes (100-run, three-fold cross-validation)	Spearman correlation + Wilcoxon rank sum + linear discriminant analysis + minimum redundancy–maximum relevance (mRMR)	15	LDA classifier
Suh et al. [33]	MRI	Yes	Yes	3D	Manual	MITK software platform	Internal	Random	Yes	LASSO	7	Logistic Regression
Vidiri et al. [28]	MRI	Yes	No	3D	Manual	3D slicer software	None	N/A	Yes	Decision tree	3	Decision tree

For each study, the radiomics workflow was evaluated, and the salient details of this process were collected. In the construction of the radiomics model, the most utilized imaging modality was CT (n = 9, 64.3%), followed by MRI (n = 4, 28.6%) and 18F-fluorodeoxyglucose positron emission tomography/computed tomography (n = 1, 7.1%). Intravenous contrast was utilized in each study. Imaging pre-processing was performed in most studies (n = 8, 57.1%). Regarding segmentation of areas of interest, this was most commonly performed in three dimensions (n = 9, 64.3%) and was most commonly performed manually (n = 10, 71.4%). A wide array of segmentation software was utilized in this process and can be found in Table 1 and Table 2.

Final Models

There was a large degree of heterogeneity among the included studies on which the modeling method was utilized. The full list of radiomics models can be found in Table 1 and Table 2; however, the most utilized models included multivariable logistic regression and least absolute shrinkage and selection operator logistic regression.

Although our primary focus was to evaluate the effectiveness of radiomics-only models in predicting HPV status, we found several studies that contained combined models incorporating both clinical and radiomics features. Five studies contained such combined models; however, our meta-analysis only assessed the diagnostic performance of radiomics models.

Most studies used a separate cohort to validate their models, using either an internal (n = 5, 35.7%), external (n = 3, 21.4%), or a combination of internal and external (n = 2, 14.3%) cohorts. Importantly, five of the models, included in four studies, did not utilize either an external or internal validation cohort. However, overall, cross-validation was performed in most studies (n = 11, 78.6%).

Diagnostic Performance

The diagnostic performance of each model was assessed using sensitivity, specificity, AUC, and accuracy (Table 3). Of the included studies, 12 models reported sensitivity, with a mean of 0.778 (standard deviation (SD) = 0.073). Similarly, 12 models reported specificity, with a mean of 0.751 (SD = 0.111). The AUC was reported by all 15 models, with a mean of 0.814 (SD = 0.081). Finally, accuracy was reported by eight models, with a mean of 0.768 (SD = 0.044).

**Table 3:** Radiomics model performance. AUC: area under the curve; CI: confidence interval

	AUC with 95% CI	Sensitivity	Specificity	AUC	Accuracy
Bagher-Ebadian et al. [31]	No CI			0.89	
Bogowicz et al. [20]	No CI	0.72	0.62	0.78	
Bos et al. [21]	0.764 (0.758–0.770)	0.76	0.71	0.764	
Choi et al. [22]	0.834 (0.738–0.930)	0.826	0.8	0.834	
Haider et al. [29]	0.75 (0.55–0.94)			0.75	
Huang et al. [23]	0.76 (0.60–0.91)	0.79	0.74	0.76	0.75
Mukherjee et al. [24]	0.80 (0.65–0.92)	0.65	0.9	0.8	0.71
Ranjbar et al. [30]	No CI	0.75	0.8	0.8	0.757
Ravanelli et al. [25]	No CI	0.833	0.926	0.944	
Ren et al. [26]	No CI	0.875	0.813	0.953	0.844
Ren et al. [26]	No CI	0.875	0.719	0.92	0.797
Sohn et al. [32]	0.744 (0.496–0.991)	0.692	0.833	0.744	0.737
Song et al. [27]	0.70 (0.62–0.79)	0.78	0.53	0.7	0.74
Suh et al. [33]	0.77 (0.50–0.96)	0.71	0.72	0.77	
Vidiri et al. [28]	No CI	0.857	0.647		0.808

Meta-Analysis of Included Studies

In total, 14 articles (15 models, given one article reported two models) reported AUC. From the 14 articles, only eight published the CIs from the AUC [21-24,27,29]. As a result, the meta-analysis was conducted on these eight studies alone.

**Figure 3:**
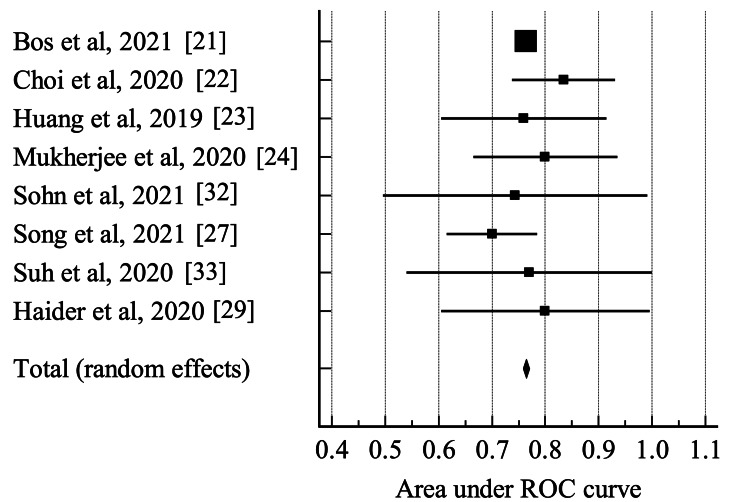
Forest plot for the area under the receiver operating characteristic (ROC) curve of the studies included in the meta-analysis.

The meta-analysis returned a pooled AUC of 0.764 (95% CI = 0.758 to 0.770). Heterogeneity of these articles was low (0%, p < 0.01). The results of the pooled area under the ROC curve can be seen in Figure 3 and Table 4. However, despite design limitations, data variability, and the complexity of HPV prediction, the current studies analyzed which aimed to predict HPV status in oropharyngeal squamous cell carcinoma showed similar diagnostic performance.

**Table 4:** Meta-analysis for pooled area under the ROC curve. ROC: receiver operating characteristic; CI: confidence interval

Study	ROC area	Standard error	95% CI	z	P	Weight (%)
						Random
Bos et al., 2021 [21]	0.764	0.00306	0.758 to 0.770			98.56
Choi et al., 2020 [22]	0.834	0.04900	0.738 to 0.930			0.39
Haider et al., 2020 [29]	0.800	0.09950	0.605 to 0.995			0.093
Huang et al., 2019 [23]	0.760	0.07910	0.605 to 0.915			0.15
Mukherjee et al., 2020 [24]	0.800	0.06890	0.665 to 0.935			0.19
Sohn et al., 2021 [32]	0.744	0.12600	0.497 to 0.991			0.058
Song et al., 2021 [27]	0.700	0.04340	0.615 to 0.785			0.49
Suh et al., 2020 [33]	0.770	0.11700	0.540 to 1.000			0.067
Total (random effects)	0.764	0.00304	0.758 to 0.770	251.401	<0.001	100.00

Quality

**Figure 4:**
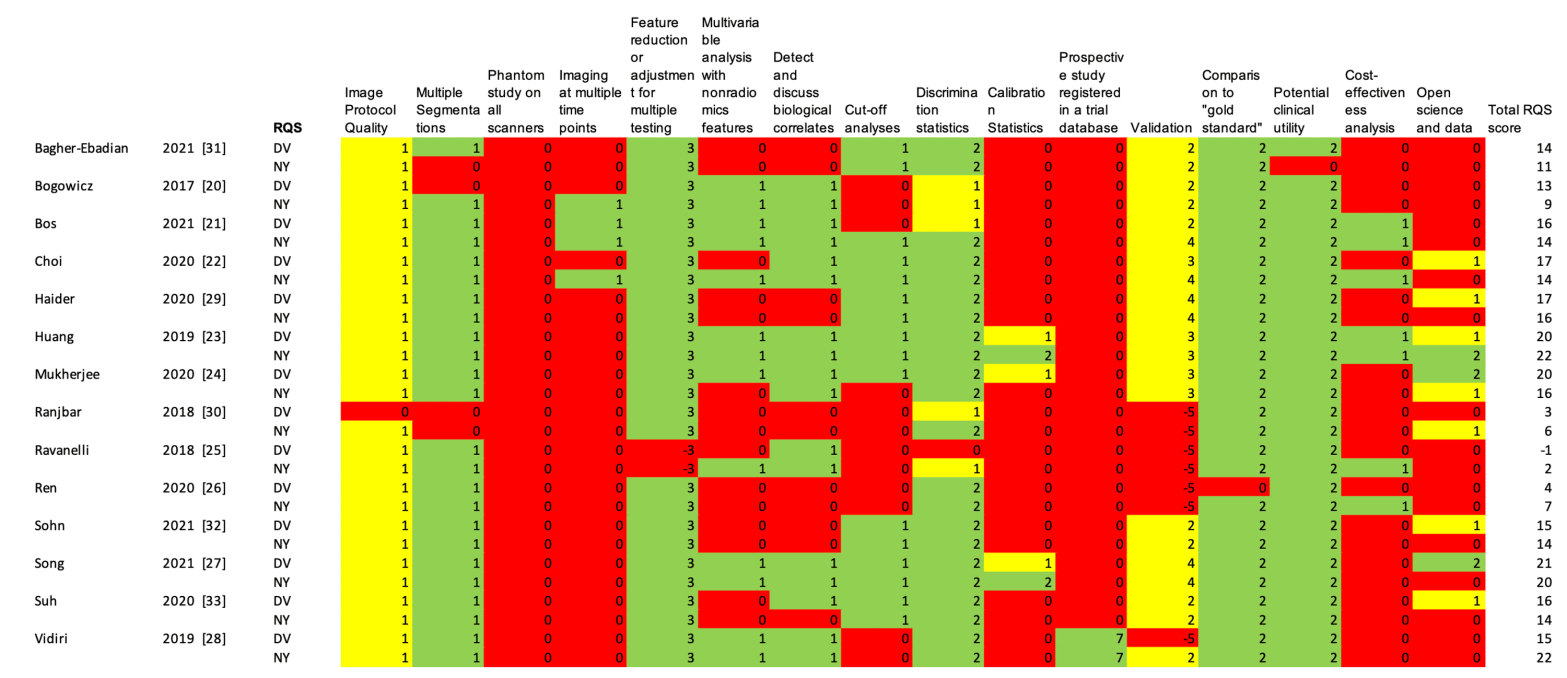
Radiomics Quality Score (RQS) of the included studies. Within each category, green represents the highest possible score and red represents the lowest possible score, with yellow representing a score somewhere between the minimum and maximum. Total possible score on the RQS is 36.

Each of the included studies was subjected to a review of the methodological quality, using the RQS, the results of which can be seen in Figure 4. In brief, the RQS is a 16-question index, with a total possible score of 36, indicating a supreme quality study. Overall, the quality of the included studies within our review varied widely, ranging from -1 to 22, with a mean of 13.4. In assessing the inter-rater reliability, the ICC was found to be 0.874, indicating good reliability [34].

Publication Bias

Publication bias was assessed as previously described using Egger’s test for a regression intercept and Begg’s test for rank correlation, as well as Deeks’ funnel plot. Begg’s test for rank correlation gave a p-value of 0.805, indicating no evidence of publication bias. Egger’s test for a regression intercept gave a p-value of 0.774, indicating no evidence of publication bias. The funnel plot demonstrated no evidence of publication bias.

Discussion

For the head and neck surgeon, particularly those involved in the care of patients with oropharyngeal carcinoma, effective and efficient techniques for the determination of HPV status are of paramount importance. The current standard of diagnosis for HPV status predominantly consists of tissue sampling and immunohistochemical testing. However, recent studies have shown promising results in discriminating between HPV-positive and negative oropharyngeal cancers using contrast-enhanced CT imaging based on radiomics analysis. This technique has several theorized advantages over the currently utilized diagnostic tools, first and foremost, the reduction of morbidity to patients. Given that all patients with squamous cell carcinoma of the oropharynx will undergo cross-sectional imaging to aid in primary diagnosis or staging, radiomics does not impose additional morbidity in the form of excess radiation via repeat imaging studies or from tissue biopsy necessary for immunohistochemical testing. Additionally, the use of machine learning algorithms, such as radiomics, may represent a less time-intensive process than traditional methods of HPV diagnosis, potentially leading to earlier decisions regarding treatment and prognosis.

Given these potential advantages of radiomics in HPV status prediction, there have been several studies published recently that seek to address its implementation in this area. In this study, we identified 14 articles that met our inclusion criteria, focusing on the use of machine learning algorithms applied to pre-treatment imaging data to predict HPV status in oropharyngeal cancer patients. We found that the overall performance of such radiomics models in predicting HPV status was good, with a mean pooled sensitivity of 0.778, specificity of 0.751, and accuracy of 0.768. When performing the meta-analysis of studies assessing the area under the ROC curve, we found that the pooled AUC was 0.764. These results indicate the effectiveness of the radiomics model in predicting HPV status, even in the absence of clinical features incorporated into the model [35].

Importantly, the results of our meta-analysis did not reveal any superiority of one model over the others included within our study regarding the prediction of HPV status. However, despite the radiomics models demonstrating modest success in predicting HPV status, these results are considerably poorer than the performance of traditional p16 immunohistochemical testing. Based on the results of a recent systematic review and meta-analysis published in 2020 by Wang et al., the combined sensitivity for p16 immunohistochemical testing was 0.94, with a combined specificity of 0.9 and an AUC value of 0.955, indicating a high diagnostic performance [36]. This is particularly important to consider in the case of oropharyngeal carcinoma, where false negatives may result in improper treatment or delays in care, ultimately resulting in increased morbidity and mortality.

Despite the overall good performance of the radiomics models in predicting HPV status with promising results, in its current form, it is not a replacement for the current mainstays of diagnosis. As such, there may be more clinical utility in radiomics in the current state as an adjunct to diagnosis or within population health to identify patterns among larger groups. One such example of the application of radiomics to population health data is in cancer screening, such as lung and breast cancer screening [37,38]. A recent review by Gillies and Schabath argues for the efficacy of radiomics in such screening techniques based on several large studies demonstrating high sensitivity, specificity, and accuracy [39]. These results indicate that such a technique may be successfully utilized in primary or secondary screening for malignancy. However, as the authors rightfully discuss, the lack of open-access data and subsequent limitations on training validation offer a substantial barrier to widespread implementation of this technique in its current form.

Additionally, radiomics, in contrast with immunohistochemical testing or other methods of HPV status determination, contains an immense degree of heterogeneity. This is evidenced by the lack of validation cohorts in several of the included studies, which significantly limits the generalizability of results for clinical practice. Furthermore, there is no consensus on the most effective methods of segmentation, feature selection, or modeling, leading to a vast number of different techniques utilized by various institutions and research groups. This issue of heterogeneity within techniques is a well-known problem within the field of radiomics, and while strides have been made to improve transparency through the use of open-source software and commonly agreed-upon principles, there still exists a large discrepancy in methodologies [18,40,41]. While this may be ameliorated in the future with continuous evolution of models and increases in funding for radiomics research, the current degree of heterogeneity and lack of sufficient validation cohorts limit its implementation in practice today.

Further evidence for the heterogeneity and discrepancy in quality among radiomics studies can be found in our RQS analysis of the included studies. Our findings demonstrated a wide array of quality among the included studies, ranging from -1 to 22 out of a possible score of 36, with a mean of roughly 13. When examining the results of the RQS for these studies, several patterns emerge. First, there was a great deal of consensus in portions of the RQS, including well-documented imaging protocols, utilizing multiple segmentations, using feature reduction, and performing discrimination statistics. However, the use of calibration statistics, discussions of cost effectiveness, and the use of open science and data were found in only a small proportion of included studies. In particular, the lack of open science and data limits the broad application of this technique and is an area of concern when considering full implementation into clinical practice.

Our study does have its limitations, which are important to discuss. First, the authors of this study do not claim to be experts on radiomics or machine learning techniques that are utilized within the individual included studies. However, utilizing two independent reviewers with consensus review by a third reviewer with more significant expertise in this domain does reduce the impact of this limitation. Additionally, it is important to note that this review included a period until June 8, 2022. In recent months, artificial intelligence has exploded in popularity through natural language processing software such as ChatGPT and other tools [42]. As such, there may be studies that have been published more recently, which would provide additional power to our study. Finally, there is a risk of heterogeneity within the articles themselves contributing to erroneous results; however, we ultimately found that the heterogeneity of the articles included for meta-analysis was low. Moreover, data sharing and algorithm sharing openly have been a limitation to enhancing models with new data.

## Conclusions

The results of our study indicate that radiomics is a potentially effective tool in the determination of HPV status for patients with oropharyngeal squamous cell carcinoma, given the diagnostic performance. This technique has numerous advantages in its non-invasive nature, along with the potential for more rapid results. However, it remains limited by the high degree of heterogeneity among current methodologies and the overall lower accuracy rates when compared to tissue sampling and immunohistochemical testing. Thus, further research is necessary before full clinical implementation of this technique.

## Figures and Tables

**Figure 5:**
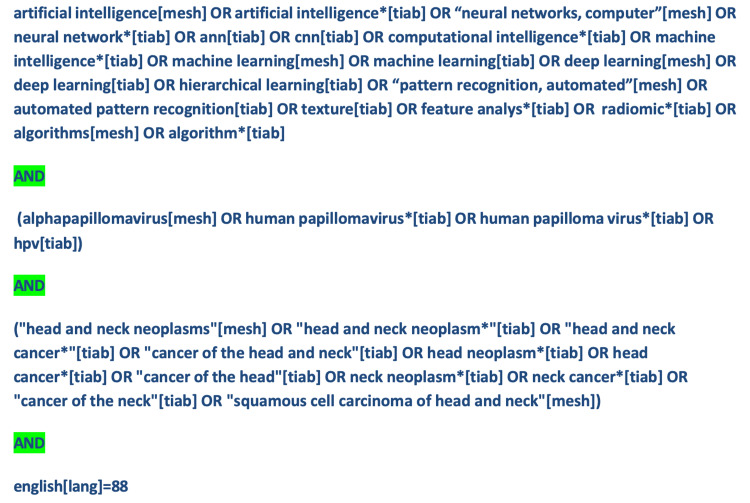
Search strategy.
